# Analysis of microarray right-censored data through fused sliced inverse regression

**DOI:** 10.1038/s41598-019-51441-0

**Published:** 2019-10-22

**Authors:** Jae Keun Yoo

**Affiliations:** Department of Statistics, Ewha Womans University, Seoul, 03760 Republic of Korea

**Keywords:** Data mining, Microarrays

## Abstract

Sufficient dimension reduction (SDR) for a regression pursue a replacement of the original *p*-dimensional predictors with its lower-dimensional linear projection. The so-called sliced inverse regression (SIR; [5]) arguably has the longest history in SDR methodologies, but it is still one of the most popular one. The SIR is known to be easily affected by the number of slices, which is one of its critical deficits. Recently, a fused approach for SIR is proposed to relieve this weakness, which fuses the kernel matrices computed by the SIR application from various numbers of slices. In the paper, the fused SIR is applied to a large-*p*-small *n* regression of a high-dimensional microarray right-censored data to show its practical advantage over usual SIR application. Through model validation, it is confirmed that the fused SIR outperforms the SIR with any number of slices under consideration.

## Introduction

Sufficient dimension reduction (SDR) in regression of *Y*|**X** **∈** *R*^*p*^ = (*X*_1_, *…*, *X*_*p*_)^T^ pursue a replacement of the original *p*-dimensional predictors **X** with its lower-dimensional linear projection without loss of information about the conditional distribution of *Y*|**X**. Equivalently, SDR seeks for finding **M** ∈ *R*^*p*×*q*^ such that

1 $$Y\coprod {\bf{X}}|{{\bf{M}}}^{{\rm{T}}}{\bf{X}},$$

where a notation of $\coprod$ represents a statistical independence and *q* ≤ *p*.

The conditional independence statement (1) indicates that the two conditional distributions of *Y* |**X** and *Y* |**M**^T^**X** are equivalent, so **X** is replaced by **M**^T^**X** with preventing loss of the information about *Y* |**X**. A subspace spanned by the columns of **M** satisfying (1) is called a dimension reduction subspace. If the subspace acquired by intersecting all possible dimension reduction subspaces is still a dimension reduction subspace, the intersection subspace is defined as the *central subspace S*_Y|X_^1^. The central subspace is minimal and unique, and its restoration is the main purpose of SDR literature. Hereafter, notations of *d* and ***η*** ∈ *R*^*p*×*q*^ represent the true dimension and orthonormal basis matrix of *S*_*Y*|**X**_, respectively. The dimension-reduced predictor ***η***^T^**X** is called sufficient predictors.

Data, whose sample size *n* is smaller than *p*, such as microarray data, high-throughput data, etc., are quite popular these days. In such data, so-called curse of dimensionality usually occurs, so a proper model-building are often problematic in practice. Then the SDR of **X** through *S*_Y|**X**_ can facilitate a model specification, so it turns out to be practically useful in such data.

One of the most popular SDR methods should be sliced inverse regression (SIR^2^). Implementation of SIR requires a categorization of a response variable *Y*, called *slicing*, and the selection of the appropriate number of slices are often critical in the application results. So far, any ideal or recommended selection guidelines to choose the number of slices are not yet known. To overcome this, a fused approach is proposed in^3^ by combining sample kernel matrices of SIR constructed by varying the numbers of slices. The combining approach in^3^ is called *fused sliced inverse regression* (FSIR). According to^3^, FSIR results in robust basis estimates of *S*_Y|**X**_ to the numbers of slices.

The purpose of this paper is to analyze a micro array right-censored survival data by implementing fused sliced inverse regression (FSIR) by^3^. The performances of FSIR will be compared with the usual SIR applications with different numbers of slices. The organization of the paper is as follows. The SIR and FSIR along with the applicability to survival regression is discussed in section 2. In the same section, the permutation dimension test is discussed. Diffuse large-B-cell lymphoma data is analyzed through SIR and FSIR, and their results are compared in section 3. We summarize our work in section 4.

We will define the following notations, which will be used frequently throughout the rest of the paper. A subspace *S*(**B**) stands for a subspace spanned by the columns of **B**. And, we define that **Σ** = cov(**X**).

## Material and Methods

### Sliced inverse regression and fused sliced inverse regression

Before explaining sliced inverse regression^2^, the predictor **X** is normalized to ${\bf{Z}}={{\boldsymbol{\Sigma }}}^{-1/2}({\bf{X}}\mbox{--}E({\bf{X}}))$. Letting *S*_*Y*|**Z**_ be the central subspace for a regression of *Y*|**Z**, then the relationship that *S*_*Y*|**X**_ = **Σ**^−1/2^*S*_*Y*|**Z**_ holds. Define *η*_*z*_ be *p* × *d* orthonormal basis matrix for *S*_*Y*|**Z**_. Consider the so-called linearity condition: (C1) *E*(**Z**|***η***_*z*_^T^**Z**) is linear in ***η***_*z*_^T^**Z**. According to^2^, a proper subspace of *S*_*Y*|**Z**_ can be constructed under linearity condition:

$$S(E({\bf{Z}}|Y))\subseteq {S}_{Y|{\bf{Z}}}\iff S({{\boldsymbol{\Sigma }}}^{-1}E({\bf{X}}|Y))\subseteq {S}_{Y|{\bf{X}}}.$$

For estimating of *S*_*Y*|**X**_ completely, it is typically assumed that *S*(**Σ**^−1^*E*(**X**|*Y*)) = *S*_*Y*|**X**_. The so-called sliced inverse regression is a method to recover *S*_*Y*|**X**_ by computing *E*(**X**|*Y*).

In population, the quantity *E*(**Z**|*Y*) should be computed without any specific assumptions on *Y* |**Z**. If *Y* is discrete with *h* levels, *E*(**Z** |*Y* = *s*) is the average of **Z** within the *s*th category of *Y*. Following this idea, if *Y* is continuous or many-valued, *Y* is transformed to a categorized response $\tilde{Y}$ with *h* levels. Then *E*(**Z**|$\tilde{Y}$ = *s*) becomes the average of **Z** within the *s*th category of $\tilde{Y}$ for *s* = 1, …, *h*. This categorization of *Y* is called *slicing*, which is done for each category to have equal numbers of observations. The SIR constructs:

$${{\rm{M}}}_{{\rm{SIR}}}={\rm{cov}}(E({\bf{Z}}|Y))\,{\rm{or}}\,{{\rm{M}}}_{{\rm{SIR}}}={\rm{cov}}(E({\boldsymbol{Z}}|\tilde{Y})).$$

In sample structure, the algorithm of SIR is as follows:

1. Construct $\tilde{Y}$ by dividing the range of *Y* into *h* non-overlapping intervals. Let *n*_*s*_ be the number of observations for the *s*th category of $\tilde{Y}$ for *s* = 1, …, *h*.
2. Compute $\hat{{E}}({\bf{Z}}|\tilde{{\rm{Y}}}={\rm{S}})={\sum }_{i\varepsilon Y=s}({\hat{{\bf{Z}}}}_{{i}}/{n}_{s})$, for *s* = 1, …, *h*, where ${\hat{{\bf{Z}}}}_{i}={\hat{\sum }}^{-1/2}({{\bf{X}}}_{{\rm{i}}}-\bar{{\bf{X}}})$.
3. Construct ${\hat{{\bf{M}}}}_{{\rm{SIR}}}$ as follows: ${\hat{{\bf{M}}}}_{{\rm{SIR}}}\hat{{\rm{c}}}\mathrm{ov}(E(\hat{{\bf{Z}}}|\tilde{Y}))={\sum }_{s=1}^{n}({n}_{s}/n)\,\hat{{\rm{E}}}({\rm{Z}}|\tilde{Y}=s)\,\hat{{\rm{E}}}{({\bf{Z}}|\tilde{Y}=s)}^{{\rm{T}}}$
4. Spectral-decompose ${\hat{{\bf{M}}}}_{{\rm{SIR}}}:{\hat{{\bf{M}}}}_{{\rm{SIR}}}={\sum }_{j=1}^{p}{\hat{\lambda }}_{j}{\gamma }_{j}{{\hat{\gamma }}_{j}}^{{\rm{T}}}$, where ${\hat{\lambda }}_{1}\ge {\hat{\lambda }}_{2}\ge \cdot \cdot \cdot \ge {\hat{\lambda }}_{p}\ge 0$.
5. Determine the structural dimension *d*. Let $\hat{d}$ denote an estimate of *d*.
6. A set of eigenvectors $({\hat{\gamma }}_{1},\ldots ,{\hat{\gamma }}_{d})$ corresponding to first $\hat{d}$ largest eigenvalues are the estimate of an orthonormal basis for *S*_*Y*|**Z**_.
7. Back-transform ${\hat{\sum }}^{-{1}/{2}}({\hat{\gamma }}_{1},\ldots ,{\hat{\gamma }}_{d})$ to have the estimate of an orthonormal basis of *S*_*Y*|**X**_.

As we can see the implementation of SIR in practice, the results may critically vary depending on the selection of *h*. This is discussed in^3^. Define that **M**_FSIR(h)_ = (**M**_SIR(1)_, …, **M**_SIR(*h*)_), where **M**_SIR(*h*)_ stands for the kernel matrix of SIR with *h* slices. Since *S*(**M**_SIR(*k*)_) = *S*_*Y*|**Z**_ for *k* = 2, 3, .., *h*, we have.

$$S({{\bf{M}}}_{{\rm{SIR}}(k)})\subseteq S({{\bf{M}}}_{{\rm{FSIR}}(h)})={S}_{Y|{\bf{Z}}},\,k=2,3,\ldots ,h.$$

In^3^, the matrix **M**_FSIR(*h*)_ is proposed as another kernel matrix to estimate *S*_*Y*|**X**_, and this approach is called *fused sliced inverse regression* (FSIR). In^3^, it is confirmed that **M**_FSIR(*h*)_ is robust to the choices of *h* through various numerical studies.

Inference on *S*_*Y*|**Z**_ is done by the spectral decomposition of ${\hat{{\bf{M}}}}_{{\rm{FSIR}}(k)}$. The eigenvectors of ${\hat{{\bf{M}}}}_{{\rm{FSIR}}(k)}$ corresponding to its non-zero eigenvalues form an estimate of an orthonormal basis of *S*_*Y*|**Z**_.

### Permutation dimension test

The true structural dimension *d* is determined by a sequence of hypothesis tests^4^. Starting with *m* = 0, test *H*_0_: *d* = *m* versus *H*_1_: *d* = *m* + 1. If *H*_0_: *d* = *m* is rejected, increment *m* by 1 and redo the test, stopping the first time *H*_0_ is not rejected and setting $\hat{d}$ = *m*. This dimension test is equivalent to testing the rank of **M**_FSIR(*h*)_. So, a proposed test statistics is as follows:

$${\hat{\Lambda }}_{m}=n{\sum }_{i=m+1}^{p}{\hat{\lambda }}_{i},$$

where $${\hat{\lambda }}_{1}\ge {\hat{\lambda }}_{2}\ge \cdot \cdot \cdot \ge {\hat{\lambda }}_{p}\ge 0$$.

Here a permutation approach is adopted to implement the dimension estimation. An advantage of the permutation test is no requirement of the asymptotics of ${\hat{\Lambda }}_{m}$. The permutation test algorithm is as follows:

1. Construct ${\hat{{\bf{M}}}}_{{\rm{FSIR}}(h)}$. Under *H*_0_: *d* = *m*, compute ${\hat{\Lambda }}_{m}$ and partition the eigenvectors: ${\hat{{\boldsymbol{\Gamma }}}}_{{1}}=({\hat{\gamma }}_{{1}},\ldots ,{\hat{\gamma }}_{{m}})\,{\rm{and}}\,{\hat{{\boldsymbol{\Gamma }}}}_{{2}}=({\hat{\gamma }}_{{m}+{1}},\ldots ,{\hat{\gamma }}_{{p}})$.
2. Construct two sets of vectors: ${\hat{\hat{V}}}_{i}\in {R}^{m\times 1}={{\hat{\Gamma }}_{{1}}}^{T}{\hat{{\rm{Z}}}}_{{i}}$ and ${\hat{U}}_{i}\in {R}^{(p-m)\times 1}={{\hat{\Gamma }}_{{2}}}^{{\rm{T}}}{\hat{{\rm{Z}}}}_{i},\,i=1,\mathrm{...},n$
3. Randomly permute index *i* of the ${\hat{U}}_{i}$ with the permuted set ${\hat{U}}_{i}^{\ast }$.
4. Construct the test statistics ${\hat{\Lambda }}_{m}^{\ast }$ based on a regression of *Y*_*i*_|(${\hat{\hat{V}}}_{i}$   ${\hat{U}}_{i}^{\ast }$).
5. Repeat steps (3–4) *N* times, where *N* is the total number of permutations. The *p*-value of the hypothesis testing is the fraction of ${\hat{\Lambda }}_{m}^{\ast }$ that exceed ${\hat{\Lambda }}_{m}$.

The setting *N* = 1000 is a widely-used choice.

### Application to survival regression

Survival regression is a study of the conditional distribution of survival time *T* given a set of predictors **X**. Naturally, SDR in the survival regression should seek for recovering the central subspace *S*_*T*|**X**_:

2 $$T\coprod {\bf{X}}|{\eta }^{T}{\bf{X}}.$$

However, since the true survival time *T* cannot be completely observed due to censoring, the direct study of *T*|**X** cannot be usually done.

Instead, the data (*Y*_*i*_, *δ*_*i*_, **X**_*i*_), *i* = 1, *…*, *n*, are collected as *n* independent and identically distributed realizations of (*T*, *C*, **X**), where *Y* = *T δ* + *C*(1 − *δ*), *δ* = 0, 1 is an indicator variable whose value is equal to 1, if *δ*(*C* > *T*) = 1 and 0, otherwise, and *C* stands for a censoring time. This type of censoring is called right-censoring. Using (*Y*_*i*_, *δ*_*i*_, **X**_*i*_), the regression of *T*|**X** is replaced as follows. The first step is a consideration of a regression of (*T*, *C*)|**X**. The construction of (*T*, *C*)|**X** directly implies that *S*_*T*|**X**_ ⊆ *S*_(*T*,*C*)|**X**_. According to^5^, the central subspace S_(*Y*,*δ*)|**X**_ from a bivariate regression of (*Y*, *δ*)|**X** is informative to *S*_*(T*,*C*)|**X**_, because *S*_(*Y*,*δ*)|**X**_ ⊆ *S*_(*T*,*C*)|**X**_. Since (*Y*, *δ*, X) are collected for survival analysis, the estimation of *S*_(*Y*,*δ*)|**X**_ can be done. The two regressions of *T*|**X** and (*Y*, *δ*)|**X** are connected in^3^ under condition: (C2) *C* $\coprod$ **X**|(***η***^T^**X**, *T*). Conditionc2 is weaker than *C* $\coprod$ (*T*, X), which is normally assumed in in survival analysis. Then, condition C2 guarantees that statement (2) is equivalent to (*T*,*C*)$\coprod$**X**|*η*^T^**X**, so we have *S*_(*T*,*C*)|**X**_ = *S*_*T*|**X**_. Therefore, the following relation directly implied:

$${S}_{(Y,\delta )|{\bf{X}}}\subseteq {S}_{(T,C)|{\bf{X}}}={S}_{T|{\bf{X}}}.$$

According to^5,6^, the equality would normally hold, because proper containment requires carefully balanced conditions. Then, SIR and FSIR are directly applicable with bivariate slicing of *Y* and *δ* to recover *S*_*T* |**X**_. Similar discussion about this can be found in section 4.2 of ^ 6^.

## Results

### Analysis of diffuse large-B-cell lymphoma data

The diffuse large-B-cell lymphoma dataset (DLBCL^7^) contains measurements of 7399 genes from 240 patients obtained from customized cDNA microarrays. For each patient, his/her survival time was recorded and varied from 0 to 21.8 years. The total uncensored cases (deceased) are 138 among 240 patients. More detailed description on the data is founded in^6–8^.

We follow the approach in^9^ to analyze the DLBCL. The DLBCL is randomly divided into the training set of 160 and the test set of 80. As usual, the training set is used for model-building, and the test set is utilized for model-validation. First, the 7399 genes in the training set, which are denoted as **X**_tr_, are initially reduced to their 40 principal components through principal component analysis. Letting $\hat{{\boldsymbol{\Omega }}}\in {R}^{7399\times 40}$ be the rotation matrix, the 40 principal components are ${\hat{{\boldsymbol{\Omega }}}}^{{\rm{T}}}{{\bf{X}}}_{{\rm{tr}}}$.

Second, the SIR is employed for the additional dimension reduction of ${\hat{{\boldsymbol{\Omega }}}}^{{\rm{T}}}{{\bf{X}}}_{{\rm{tr}}}$ with observed survival time and censoring status as bivariate responses. Let $\hat{{\bf{B}}}\in {R}^{40\times d}$ stand for the estimated matrix. According to^9^, the dimension *d* is estimated to be one. The finalized estimated sufficient predictors through this two-step dimension reduction are denoted as ${\hat{{\boldsymbol{\eta }}}}^{{\rm{T}}}{{\bf{X}}}_{{\rm{tr}}}$ with $\hat{{\boldsymbol{\eta }}}\in {R}^{7399\times 1}=\hat{{\boldsymbol{\Omega }}}\hat{{\bf{B}}}{\rm{.}}$

For model-building, the Cox-proportional hazards model was fitted with ${\hat{{\boldsymbol{\eta }}}}^{{\rm{T}}}{{\bf{X}}}_{{\rm{tr}}}$. For model-validation, the predicted scores and the corresponding area under ROC curves for prediction of survival time from 1 to 10 years for both the training and test sets were computed. For the test set, the dimension- reduced predictors are defined as ${\hat{{\boldsymbol{\eta }}}}^{{\rm{T}}}{{\bf{X}}}_{{\rm{te}}}$, where $\hat{{\boldsymbol{\eta }}}$ is obtained from the training set and **X**_te_ stands for the predictors in the test set. The area closer to one indicates better estimation.

**Figure 1:**
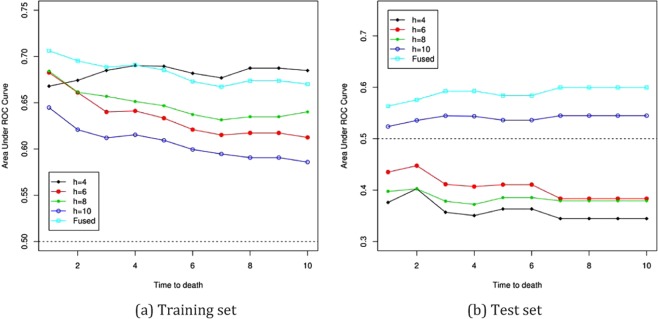
Area under ROC curves at time 1 to 10 years for DLBCL data in Section 3: *h* = 4, 6, 8, 10, sliced inverse regression with the according number of slices; Fused, fused sliced inverse regression with *h* = 10.

One potentially arguable issue in the analysis in the context should arise on the selection of the number of slices *h* in the SIR application. As discussed in the previous section, its performance inevitably depends on *h*. To investigate how serious they impact on the model-validation, we consider *h* = 4, 6, 8 and 10 for SIR along with FSIR. Following the guidance of ^3^, 10 slices are used in FSIR. The area under ROC curve for the training and test sets are reported in Fig. 1.

First, we see the areas under ROC curves for the training set in Fig. 1(a). Larger areas indicate better prediction performances. For the SIR application, the smaller numbers of slices show the better performances. The FSIR is not best among the all application of SIR considered here, but there are no notable differences to the best results, which is with *h* = 2, among all the SIR applications. Therefore, for the training set, the FSIR is not cause of concern at all. In the case of the test set in Fig. 1(b), the FSIR shows better prediction performances than any of the SIR applications. The prediction results by the FSIR is consistent in both the training and test sets, while the usual SIR applications are very sensitive to the choices of *h*, as expected. The application of the FSIR to the data is concluded to be successful.

## Discussion

According to Fig. 1(a,b), the areas under ROC curves for the training and test sets are reversed against *h* in the SIR applications. In the training set, smaller numbers of slices have larger areas, while the areas with smaller numbers of *h* become smaller in the test sets, which is even below 0.5. The area equal to 0.5 is often used as the cut-off. Therefore, for SIR, the application with *h* = 10 alone is above 0.5 in both train and test sets, although its performance is worst among the others in the train set. The FSIR, however, shows reliable and consistently good performances in both training and test sets.

The best selections of *h* in the training set and the test set are different, and this selection bias in *h* can cause the ironic results in SIR. This bias also affects the estimation of *h* in the analysis. With level 5%, the SIR application with *h* = 4 and 8 determines that $\hat{d}$ = 0 with the corresponding *p*-values of 0.139 and 0.244 for *H*_0_: *d* = 0, respectively. However, the SIR with *h* = 6 and 10 determines that $\hat{d}$ = 1 (*h* = 6: 0.009 for *H*_0_: *d* = 0 and 0.097 for *H*_0_: *d* = 1 & *h* = 10: 0.007 for *H*_0_: *d* = 0 and 0.10 for *H*_0_: *d* = 1). This confirms the severe sensitivity of the SIR to the selection of *h* in the high-dimensional data analysis. The FSIR determines that $\hat{d}$ = 1 with the *p*-values of 0.014 for *H*_0_: *d* = 0 and of 0.115 for *H*_0_: *d* = 1. This shows that the FSIR has potential advantages over the SIR in high-dimensional data analysis in practice.

## Conclusion

Fused sliced inverse regression (FSIR) proposed by^3^ solves the sensitiveness of slice inverse regression (SIR^2^) to the number of slices by combining SIR kernel matrices. In this paper, the fused sliced inverse regression is applied to high-dimensional microarray right-censored data to show the potential advantage to large *p*-small *n* data over the usual SIR application. The predictors are initially reduced through principal components analysis, and then SIR and FSIR are implemented with 40 principal components. According to model-validation, the SIR reveals its sensitiveness to the number of slices. Moreover, ironic validation results are observed in the training and test sets. For SIR, the numbers of slices to have better performances in the training set show worse performances in the test set. This may be because good slicing schemes in the training set do not coincide with that in the test set. This is confirmed again through the estimation of the true structural dimension. However, FSIR shows better performances in the training and test sets than all SIR-application under consideration. This proves a practical advantage of FSIR over SIR.

The usage of FSIR can improve the accuracy in high-dimensional data analysis, which often arise in many scientific fields including biological sciences, so it can contribute to discover new founding in the many science areas.

## Data Availability

The dataset of the diffuse large-B-cell lymphoma dataset (DLBCL^7^) is available at the following two web locations: http://llmpp.nih.gov/DLBCL; http://statweb.stanford.edu/~tibs/superpc/staudt.html.
